# Feasibility of identifying important changes in care management resulting from cardiovascular magnetic resonance (CMR) using hospital episode data in patients who activate the primary percutaneous coronary intervention (PPCI) pathway

**DOI:** 10.1186/s12874-019-0755-3

**Published:** 2019-06-06

**Authors:** Maria Pufulete, Jessica Harris, Stephen Dorman, Lynn Cook, Chiara Bucciarelli-Ducci, John Greenwood, Richard Anderson, Rachel Brierley, Barnaby C. Reeves

**Affiliations:** 1 Clinical Trials and Evaluation Unit, University of Bristol, Level 7, Bristol Royal Infirmary, Queen’s Building, Bristol, UK; 2 NIHR Bristol Cardiovascular Research Unit, Bristol Heart Institute, University of Bristol, Bristol, UK; 3 Department of Information Management & Technology, University Hospitals Bristol NHS Foundation Trust, Bristol, UK; 4 Multidisciplinary Cardiovascular Research Centre and Leeds Institute of Cardiovascular and Metabolic Medicine, University of Leeds, Leeds, UK; 5 University Hospitals of Wales, Heath Park, Cardiff, UK

**Keywords:** Cardiovascular magnetic resonance (CMR), Primary percutaneous coronary intervention (PPCI), Hospital Episode Statistics (HES), England, Patient episode database Wales (PEDW)

## Abstract

**Background:**

We determined whether it is feasible to identify important changes in care management resulting from cardiovascular magnetic resonance (CMR) in patients who activate the primary percutaneous coronary intervention (PPCI) pathway from hospital episode data, in order to construct a composite primary outcome (hypothesised to reduce the risk of major adverse cardiac-related events, MACE) to compare patients exposed to CMR or not.

**Methods:**

We used Hospital Episode Statistics (HES) and Patient Episode Database for Wales (PEDW) to identify clinical events that reflected important changes in management in the year following the index admission in five subgroups of patients who activated the PPCI pathway recruited as part of a feasibility cohort study (*n* = 1655 with HES/PEDW data). For all subgroups, we identified frequency of events and time to the first event for each change in management.

**Results:**

We identified all clinical events (new diagnoses, additional diagnostic tests and procedures) except for medication prescriptions. Diagnostic tests were underestimated because most are carried out in outpatient clinics and outpatient datasets had missing procedure codes for 74% of patients (some tests done in hospital may also not be recorded). We successfully tabulated frequencies of events and distributions of times to first event for most changes in management by CMR status and in CMR / non CMR centres.

**Conclusions:**

It is feasible to identify changes in care management between patients who have / do not have CMR within relevant patient subgroups. Further work to derive a weighting algorithm is required before attempting to combine the events in a composite endpoint.

**Electronic supplementary material:**

The online version of this article (10.1186/s12874-019-0755-3) contains supplementary material, which is available to authorized users.

## Background

Prospective patient registries provide a real-world view of clinical practice and patient outcomes including safety (http://www-new.njrcentre.org.uk/njrcentre/Home/tabid/36/Default.aspx) [1–4]. They can also be useful to estimate comparative effectiveness providing that they can accrue sufficient data quickly enough to estimate treatment effects with adequate precision. In patients who have attended an Emergency Department (ED) with ST-elevation myocardial infarction (STEMI), the frequency of subsequent major adverse cardiac-related events (MACE) has decreased markedly over the past 20 years [5–8]. This decrease has been achieved mainly through improvements in management and use of evidence-based pharmacological and interventional therapies. Therefore, studies evaluating the effectiveness of alternative management pathways in STEMI populations using MACE as the primary outcome require large sample sizes to achieve satisfactory power.

We conducted a study to determine the feasibility of setting up a registry linking data collected during usual care to assess the clinical and cost-effectiveness of cardiovascular magnetic resonance (CMR) in patients who activate the primary percutaneous coronary intervention (PPCI) pathway [9]. Although a long-term objective of the registry would be to compare the incidence of MACE in patients who do or do not have CMR after the index event, the relatively low frequency of MACE after PPCI in this population (about 13% [9]) means that the study would have to accrue over 27,000 subjects to detect a clinically important (assumed to be 10%) relative reduction in the incidence of MACE with 90% power. Therefore, a key objective of the feasibility study was to define a primary composite outcome, acceptable to cardiologists and other stakeholders (e.g. healthcare providers) as representing a clinically important change in management (e.g. expected to prevent future MACE and change practice/commissioning) resulting from CMR that could be used for the registry in the medium term.

We identified important changes in management resulting from CMR (and the specific patient subgroups these changes in management relate to) using formal consensus methods [10]. In this paper, we report on whether it is feasible to identify these changes in management during follow up from routinely collected hospital episode data (Hospital Episode Statistics, HES; Patient Episode Database Wales, PEDW). HES is commonly used for outcome ascertainment in cardiovascular disease populations and has been shown to be valid and reliable in both registries and clinical trials [11–14]. If successful, this process of ascertainment could be used to define a primary composite outcome to compare groups of PPCI patients exposed to CMR or not.

## Methods

### Patient recruitment for the feasibility study

Patients were recruited from four NHS hospitals in England and Wales with 24/7 PPCI services (Bristol, Leeds, Swansea and Cardiff) between May 2013 and September 2014. Two hospitals (Bristol and Leeds) were defined as “CMR centres”, i.e. hospitals that had a local dedicated CMR service; the other two (Swansea and Cardiff) were defined as “non-CMR centres”, i.e. hospitals without access to a local dedicated CMR service. Patient identification, recruitment and consent have been described previously [9]. Patients entered the cohort at the point they activated the PPCI pathway (i.e. had an emergency angiogram), regardless of whether PPCI was carried out or not.

### Formal consensus to identify important changes in management (and the subgroups of patients to which these referred)

We used formal consensus (literature review and cardiologist expert opinion) to formulate consensus statements about important changes in management arising from the use of CMR in patients who activate the PPCI pathway. The formal consensus process has been described elsewhere [10]. Five patient subgroups were identified as potentially benefitting from CMR, namely patients who: i) have an out of hospital cardiac arrest (OHCA); ii) have “normal” (unobstructed) coronary arteries; iii) develop LV thrombus after STEMI; iv) have multivessel disease (MVD, defined as 2 or more vessels with > 50% stenosis pre-PCI); and v) underwent PPCI and have a suspected poor prognosis (assumed to be identifiable from CMR imaging biomarkers).

### Identifying the patient subgroups defined through formal consensus

Three of the patient subgroups (patients who underwent PPCI, patients with unobstructed coronary arteries and patients with MVD) were defined using data collected at cohort entry (related to the index admission). Two subgroups, patients who had had an OHCA and those who went on to develop LV thrombus, could only be identified from HES / PEDW (data collected at cohort entry relating to the presence or absence of these conditions were missing for a large proportion of participants), using ICD-10 diagnosis codes I46 “Cardiac arrest” and I23.6 “Thrombosis of atrium, auricular appendage and ventricle as current complications following acute myocardial infarction”, respectively.

### Identifying CMR exposure

CMR exposure was defined as documentation of CMR in the imaging dataset (collected from “CMR centres”) within 10 weeks of their index admission; this time frame was chosen to capture both urgent and non-urgent (out-patient) CMR scans.

### Identifying the key clinical “events” that reflected the important changes in management

Initially, cardiologist members of the clinical team identified the key clinical “events” that they expected to reflect the important changes in management characterised through the consensus process. We then used HES and PEDW inpatient and outpatient data to ascertain these changes in management, compiling a list of diagnosis and procedure codes (International Classification of Disease, ICD-10; Office of Population Censuses and Surveys Classification of Interventions and Procedures, OPCS) representing the key clinical events with the help of a clinical coder. Most changes in management were defined by Boolean combinations of multiple ICD-10 / OPCS codes; for example, the “use of additional diagnostic tests during follow-up” was ascertained by OPCS codes for echocardiography (K58.5 OR U20.1 OR U20.2 OR U20.3 OR U20.4 OR U20.5), single photon emission computed tomography (SPECT) (U21.4); positron emission tomography (PET; U10.4 OR U21.3 OR U36.2), intra vascular ultrasound (IVUS; K51.2 OR L726), pressure-wire (K63.4 OR K63.5 OR K63.6) AND K51.8 AND Y44.2 AND Y53), radionuclide angiocardiography (U10.5) or computed tomography angiography (U10.2). We identified the key clinical events relating to the changes in management for each patient subgroup separately. The full code list used to identify changes in management is shown in the Additional file 1. For those patients who underwent CMR, any outpatient appointments that occurred between the index admission and the date of CMR were excluded. We did not use Accident and Emergency (A&E) data because most of the changes in management identified in the formal consensus process were deemed unlikely to have occurred in the A&E setting and diagnoses and procedures are not well coded in the A&E dataset.

### Statistical analysis

Data were analysed using Stata/IC (V13, StataCorp LP, Texas, USA). The analysis was descriptive, with quantitative results expressed as counts and percentages. We calculated the frequencies of all events representing changes in management (e.g. number of new diagnoses, additional diagnostic tests, number / rate of outpatient appointments, etc.) in patients who did / did not receive CMR for each patient subgroup in the 12 months following the index admission.

We also calculated the time to first event (in participants in whom an event occurred) for each change in management (e.g. time to first new diagnosis, time to the first diagnostic test, time to next revascularisation in patients with multivessel disease, etc.) and summarised these with medians and interquartile ranges (IQR). Dates of events were available for all events, which allowed us to describe the time to first event for each change in management and for all subgroups, and to visualise the data using Kaplan Meier curves and other time to event plots. We did not compare differences between patients who did / did not receive CMR in any of the subgroups because this was not an objective; we were primarily interested in the feasibility of identifying relevant events and the frequencies of events in most subgroups were small. We did not attempt to derive a composite outcome because further research would be required to derive an appropriate weighting algorithm to combine the different clinical events.

## Results

We recruited 1670 patients across the four hospitals, of whom 1655 (99%) had HES/PEDW data. Of these, 89% of patients underwent PPCI; 11% were found to have unobstructed coronary arteries, 44% of patients had MVD, 7% had out-of-hospital cardiac arrest (OHCA) and 0.06% had left ventricular (LV) thrombus. The proportions of patients in each subgroup were similar between hospitals, except for patients with MVD (19% in hospital D vs. 48, 51 and 53% in hospitals A, B and C, respectively) (Table 1).

**Table 1:** Frequency of patient subgroups by hospital

	Hospital A (*n* = 758)	Hospital B (*n* = 272)	Hospital C (*n* = 316)	Hospital D (*n* = 309)	Total (*n* = 1655)
PPCI	651 (86%)	246 (90%)	291 (92%)	279 (90%)	1467 (89%)
MVD	361 (48%)	138 (51%)	169 (53%)	58 (19%)	726 (44%)
LV thrombus after PPCI	1 (0.13%)	0	0	0	1 (0.06%)
Unobstructed coronary arteries	107 (14%)	26 (10%)	25 (8%)	30 (10%)	188 (11%)
OHCA	57 (8%)	22 (8%)	16 (5%)	14 (5%)	109 (7%)

Percentages do not add up to 100 because some patients were in more than one subgroup

*LV* left ventricular, *MVD* multivessel disease, *OHCA* out of hospital cardiac arrest, *PPCI* primary percutaneous coronary intervention

### Identifying changes in management in hospital episode statistics data

The ICD-10 diagnosis codes and OPCS procedure codes of the clinical events representing important changes in management resulting from CMR are shown in Table 2**.** We successfully identified the events described in the table, except for medications; these are not available in HES/PEDW data. We were also unable to obtain data about medications on discharge for a large proportion of the cohort. Therefore, we could not identify changes in medication prescribed during follow up. All relevant non-ischaemic diagnoses were identifiable in admitted patient care data, as were procedures such as implantation of devices, repeat revascularisation and additional diagnostic tests. In contrast, we could not identify any of these from outpatient care data because ICD-10 diagnosis codes and OPCS procedure codes associated with each outpatient episode were missing for a large proportion of patients. All outpatient visits should have a diagnostic code associated with the visit. However, for at least one of their visits, 25% of patients had missing ICD-10 diagnosis codes, 74% of patients had missing OPCS procedure codes (which may indicate either that no procedure took place or that a procedure took place but was not recorded), and 74% of patients had unknown “Unknown and unspecified causes of morbidity” as their ICD-10 diagnosis code).

**Table 2:** Data sources and definitions used for identifying changes in management up to 12 months after the index admission

Important change in management resulting from CMR^a^	Patient subgroup	Data source	ICD-10 diagnosis codes and OPCS procedure codes (up to 12 months after the index admission)
New diagnosis (non-ischaemic)	Unobstructed coronary arteries OHCA	HES / PEDW admitted patient care data	Any record of the following: − Takotsubo cardiomyopathy (I42.8 AND F43.8) − Myocarditis (I51.4) − Pericarditis (I30 OR I31.0 OR I31.1 OR I31.9 OR I32.0 OR I32.1 OR I32.8 OR I01.0 OR I02.0 OR I09.2) − Endocarditis (I33.9) − Coronary spasm (I20.1)
Changes in medication	PPCI MVD LV thrombus after PPCI Unobstructed coronary arteries OHCA	Not available	Not available
Additional diagnostic tests	PPCI MVD LV thrombus after PPCI Unobstructed coronary arteries OHCA	HES / PEDW admitted patient care data and outpatient care data	Any record of the following: − Echocardiography (K58.5 OR U20.1 OR U20.2 OR U20.3 OR U20.4 OR U20.5) − Single photon emission computed tomography (SPECT) (U21.4) − Positron emission tomography (PET) scans (U10.4 OR U21.3 OR U36.2) − Intravascular ultrasound (IVUS) (K51.2 OR L726) − Pressure wire ((K63.4 OR K63.5 OR K63.6) AND K51.8 AND Y44.2 AND Y53) − Radionuclide angiocardiography (U10.5) − Computed tomography angiography (U10.2)
Implantation of devices	PPCI OHCA	HES / PEDW admitted patient care data	Any record of the following: − Cardiac resynchronization therapy (CRT) (K60.7 OR K61.7 OR K59.6) − Implantable cardioverter defibrillator (ICD) (K59 OR K72)
Revascularisation (PCI or CABG) within 3 months	MVD	HES / PEDW admitted patient care data	Any record of the following (up to 3 months after the index admission): − PCI (K49 OR K50 OR K75) − CABG (K40 OR K41 OR K42 OR K43 OR K44 OR K45 OR K46)
Frequency of cardiology outpatient appointments	PPCI MVD LV thrombus after PPCI Unobstructed coronary arteries OHCA	HES / PEDW outpatient care data	Rate of outpatient visit attended (count of visits divided by follow-up time) where the treatment speciality in which the consultant responsible was working during the period of care was Cardiology (code = 320).

*CMR* cardiovascular magnetic resonance, *HES* Hospital Episode Statistics, *ICD-10* International Classification of Diseases, *LV* left ventricular, *OHCA* out of hospital cardiac arrest, *MVD* multivessel disease, *OPCS* Office of Population Census and Surveys Classification of Intervention and Procedures, *PEDW* Patient Episode Database Wales, *PPCI* primary percutaneous coronary intervention

^a^Identified in formal consensus

### Important changes in management by CMR status and in CMR / non CMR centres

**Fig. 1:**
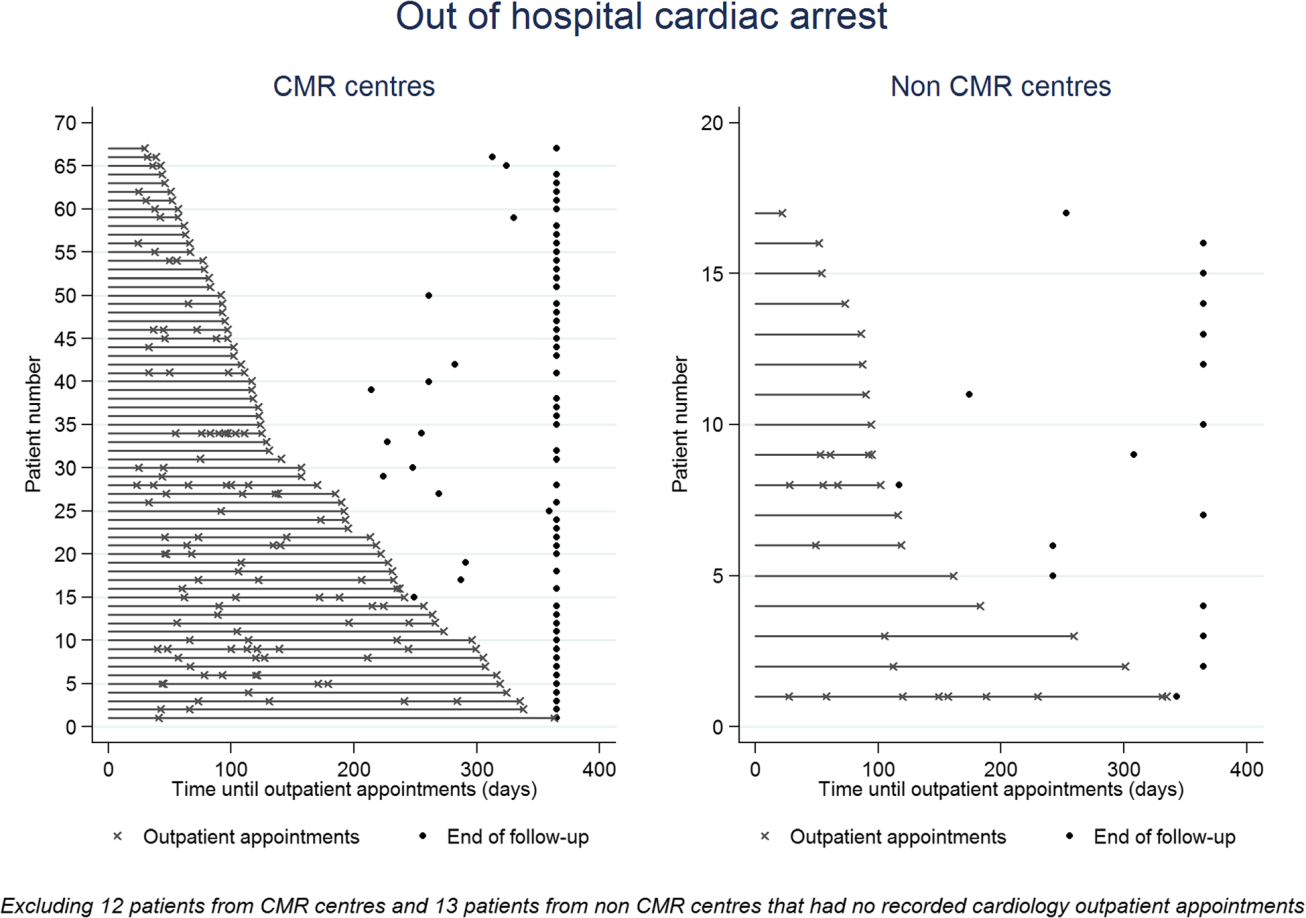
Number of cardiology outpatient appointments up to 12 months after the index event and time to each appointment for patients in the OHCA subgroup by CMR vs non CMR centre

**Fig. 2:**
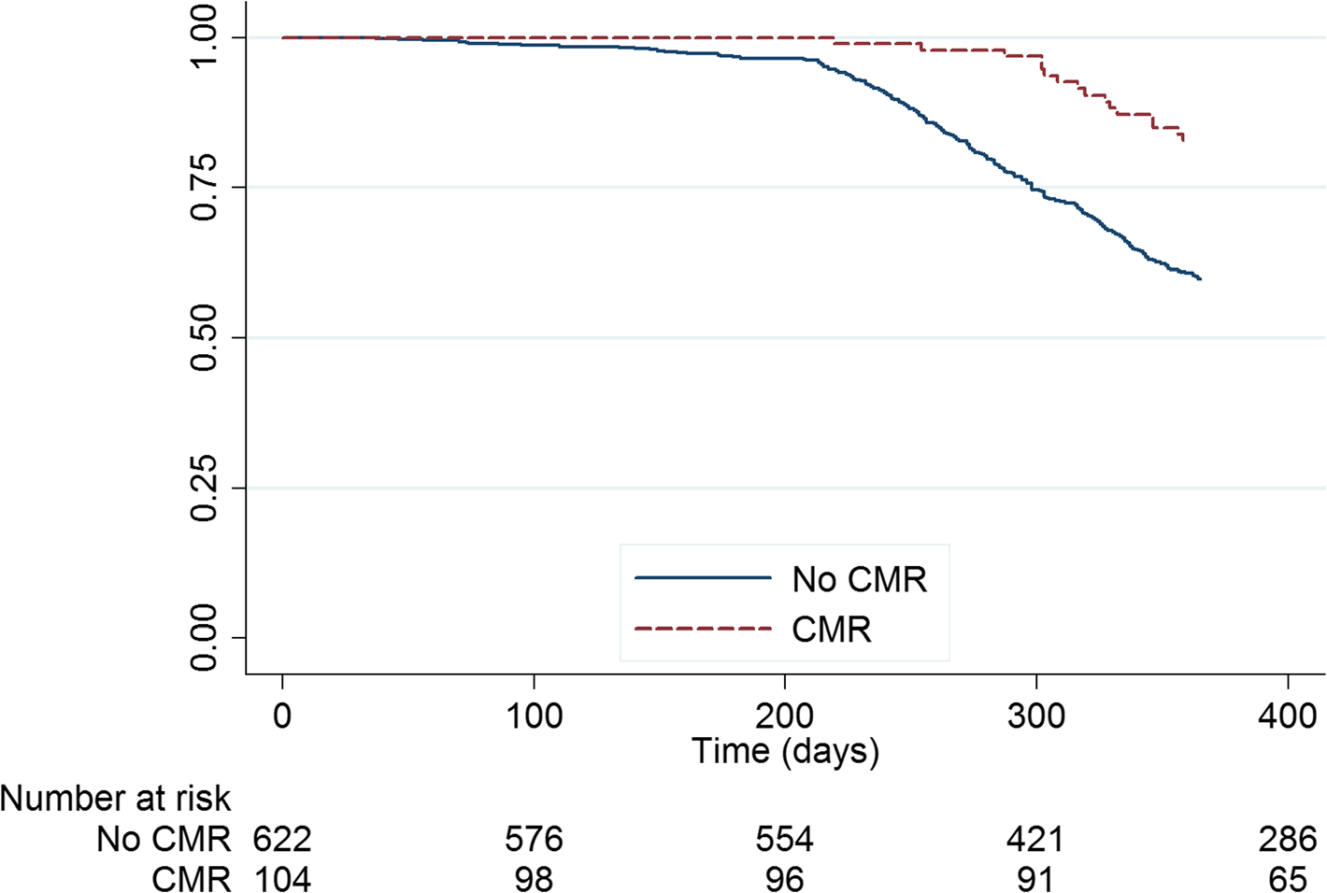
Kaplan-Meier curve for time to first additional diagnostic test in the 12 months after the index event for patients in the MVD subgroup by CMR status

Tables 3 and 4 show the number of events and time to first event representing changes in management in the relevant patient subgroups identified in HES / PEDW up to 12 months after the index admission by CMR status and in CMR vs non CMR centres, respectively. It was possible to observe differences in both the frequency of events and time to first event by CMR status or in CMR vs non CMR centres. For example, across all subgroups, patients in CMR centres had more outpatient appointments and an earlier time to first appointment (see Fig. 1 for an example in the OHCA patient subgroup), and more frequent diagnostic tests and implantation of devices than patients in non CMR centres. Across most patient subgroups, patients in CMR centres and patients who had CMR seemed to have had an admission for an additional diagnostic test later than patients in non-CMR centres or patients who did not have CMR, respectively (see Fig. 2 for an example of CMR vs no CMR in the MVD patient group).

**Table 3:** Events and time to first event representing changes in management identified in HES / PEDW inpatient and outpatient data up to 12 months after the index admission by CMR status

	Number of events (%) identified in HES / PEDW up to 12 months after the index admission and number of days (median and IQR) until the first event after the index admission										
Patient subgroup	New diagnoses (e.g. Takotsubo, myocarditis, pericarditis, endocarditis, coronary spasm)		Changes in medication	Additional diagnostic tests (ECHO, SPECT, PET, IVUS, pressure-wire, radionuclide angiocardiography, CT angiography)		Implantation of devices (CRT or ICD)		Revascularisation (PCI or CABG) within 3 months		Cardiology outpatient appointments (median, IQR)	
	No. of events	No. of days		No. of events	No. of days	No. of events	No. of days	No. of events	No. of days	No. of events	No. of days
PPCI			No data available					N/A			
CMR (*n* = 152)	0	–		16 (11%)	107 (50, 240)	0	–			2 (1, 3)	64 (34, 99)
No CMR (*n* = 1312)	10 (1%)	16 (8, 20)		129 (10%)	74 (27, 171)	6 (0.5%)	147 (48, 171)			1 (1, 2)	55 (33, 95)
MVD											
CMR (*n* = 104)	0	–		11 (11%)	85 (50, 221)	0	–	11 (11%)	99 (50, 180)	2 (1, 3)	76 (52, 116)
No CMR (*n* = 622)	4 (1%)	13 (8, 110)		61 (10%)	62 (24, 130)	4 (1%)	147 (84, 169)	64 (10%)	66 (39, 122)	1 (1, 2)	57 (33, 95)
LV thrombus after PPCI								N/A			
CMR (*n* = 1)	0	–		0	–	0	–			3 (3, 3)	66 (66, 66)
No CMR (*n* = 0)	0	–		0	–	0	–			–	–
Unobstructed coronary arteries											
CMR (*n* = 35)	1 (3%)	358 (358, 358)		6 (17%)	93 (58, 140)	1 (3%)	78 (78, 78)			2 (1, 3)	67 (52, 121)
No CMR (*n* = 139)	1 (1%)	180 (180, 180)		12 (9%)	123 (78, 284)	3 (2%)	245 (71, 262)			1 (0, 2)	67 (44, 106)
OHCA											
CMR (*n* = 14)	0	–		2 (14%)	110 (79, 141)	0	–			1 (1, 3)	65 (61, 104)
No CMR (*n* = 95)	1 (1%)	48 (48, 48)		10 (11%)	62 (48, 93)	5 (5%)	126 (71, 167)			1 (1, 2)	64 (41, 99)

*CABG* coronary artery bypass grafting, *CMR* cardiovascular magnetic resonance imaging, *CRT* cardiac resynchronisation therapy, *ECHO* echocardiography, *HES* Hospital Episode Statistics, *ICD* implantable cardioverter defibrillator, *IVUS* intravascular ultrasound, *IQR* interquartile range, *LV* left ventricular, *MVD* multivessel disease, *OHCA* out of hospital cardiac arrest, *PET* positron emission tomography, *PCI* percutaneous coronary intervention, *PEDW* Patient Episode Database Wales, *PPCI* primary percutaneous coronary intervention

**Table 4:** Events and time to first event representing changes in management identified in HES / PEDW inpatient and outpatient data up to 12 months after the index admission in CMR vs. non CMR centres (hospitals A and B vs. hospitals C and D, respectively)

	Number of events (%) identified in HES / PEDW up to 12 months after the index admission and number of days (median and IQR) until first event after the index admission										
Patient subgroup	New diagnoses (e.g. Takotsubo, myocarditis, pericarditis, endocarditis, coronary spasm)		Changes in medication	Additional diagnostic tests (ECHO, SPECT, PET, IVUS, pressure-wire, radionuclide angiocardiography, CT angiography)		Implantation of devices (CRT or ICD)		Revascularisation (PCI or CABG) within 3 months		Cardiology outpatient appointments (median, IQR)	
	No. of events	No. of days		No. of events	No. of days	No. of events	No. of days	No. of events	No. of days	No. of events	No. of days
PPCI			No data available					N/A			
CMR centres (*n* = 897)	6 (1%)	14 (8, 17)		112 (12%)	79 (33, 205)	5 (1%)	167 (126, 171)			2 (1, 3)	46 (30, 81)
Non CMR centres (*n* = 567)	4 (1%)	20 (14, 34)		33 (6%)	45 (24, 116)	1 (0.2%)	48 (48, 48)			1 (0, 1)	90 (57, 126)
MVD											
CMR centres (*n* = 499)	4 (1%)	13 (8, 110)		61 (12%)	69 (32, 159)	4 (1%)	147 (84, 169)	53 (11%)	71 (41, 151)	2 (1, 3)	52 (32, 87)
Non CMR centres (*n* = 227)	0	–		11 (5%)	59 (15, 122)	0	–	22 (10%)	63 (43, 88)	1 (0, 1)	95 (68, 136)
LV thrombus after PPCI								N/A			
CMR centres (n = 1)	0	–		0	–	0	–			3 (3, 3)	66 (66, 66)
Non CMR centres (*n* = 0)	–	–		–	–	–	–			–	–
Unobstructed coronary arteries											
CMR centres (*n* = 133)	2 (2%)	269 (180, 358)		16 (12%)	103 (68, 205)	4 (3%)	162 (75, 254)			1 (0, 2)	65 (46, 103)
Non CMR centres (*n* = 41)	0	–		2 (5%)	184 (69, 299)	0	–			0 (0, 1)	89 (54, 133)
OHCA											
CMR centres (*n* = 79)	0	–		9 (11%)	79 (62, 141)	4 (5%)	147 (99, 169)			2 (1,3)	63 (42, 95)
Non CMR centres (*n* = 30)	1 (3%)	48 (48, 48)		3 (10%)	48 (5, 74)	1 (3%)	48 (48, 48)			1 (0,1)	86 (52, 105)

*CABG* coronary artery bypass grafting, *CMR* cardiovascular magnetic resonance imaging, *CRT* cardiac resynchronisation therapy, *ECHO* echocardiography, *HES* Hospital Episode Statistics, *ICD* implantable cardioverter defibrillator, *IVUS* intravascular ultrasound, *IQR* interquartile range, *LV* left ventricular, *MVD* multivessel disease, *OHCA* out of hospital cardiac arrest, *PET* positron emission tomography, *PCI* percutaneous coronary intervention, *PEDW* Patient Episode Database Wales, *PPCI* primary percutaneous coronary intervention

## Discussion

### Main findings

We have shown that it is feasible to identify clinical events expected to prevent future MACE and describe their frequencies and timing in relation to index PPCI pathway activation using only routinely collected NHS hospital episode data. We were able to identify relevant patient subgroups specified previously by combining data about the index event, which was obtained directly from hospitals, with admitted patient care (APC) hospital episode data. For each patient subgroup, we listed the “key clinical events” (e.g. new diagnoses or procedures, additional diagnostic tests, changes in medication, outpatient appointments) that reflected a change in management, identified through discussions with relevant clinicians. By looking at care pathways, where available, we confirmed that we captured all aspects of patient management in these key clinical events. We then compiled a list of the relevant ICD-10 diagnosis codes and OPCS procedure codes, identified these codes in hospital episode data for participants up to 12 months following the index procedure, and tabulated the events by patient subgroup.

We considered both the frequency of the events representing important changes in management and time to the first event for a composite primary outcome. This is because, for some changes in management (e.g. additional revascularisation for MVD patients or a new diagnosis in patients with unobstructed coronary arteries), it is the time to the event that represents the main change in management resulting from CMR. For other changes in management (e.g. additional diagnostic tests in patients with unobstructed coronaries), it is a change in the frequency/rate of an event this would represent the main change in management (e.g. a patient receiving fewer tests or outpatient appointments). We were able to tabulate the frequencies of events and their timing, and visualise these by CMR status (at the patient and centre level) for all patient subgroups. We were able to observe differences in the process of healthcare in all patient subgroups by CMR status. We did not explore possible reasons for apparent differences since this was not the aim of our study; the feasibility of implementing the consensus statements using hospital episode data was our main objective.

Composite endpoints combine several events of interest within a single outcome variable. A composite endpoint is intended to increase the frequency of a binary primary outcome and thereby increase the power of a study for a given relative treatment effect (e.g. risk, odds or hazard ratio). We did not attempt to combine clinical events to derive a composite primary outcome, either by subgroup or overall, mainly because we had no basis on which to weight different events. The derivation of a weighting algorithm to incorporate the “importance” of different clinical events would require further research, possibly involving a formal consensus process with clinicians, commissioners and patients, in order to provide a rationale for its use in a future registry assessing the clinical effectiveness of CMR in patients who activate the PPCI pathway. Another difficulty is that clinical events were characterised by different metrics (frequencies and rates) in our study, so some changes in management (e.g. the rate of cardiology outpatient appointments or additional diagnostic tests; changes in medication) cannot easily be included in a composite outcome for a time-to-event model.

### Strengths and limitations

Our study has several strengths. It is based on important changes in management and hospital activities previously identified systematically using formal consensus methodology, with input from a range of all stakeholders (clinicians, methodologists and HES coders). By using routinely collected hospital data, the study benefits from an efficient design and avoids the use of primary data collection. Our decision to use HES/PEDW data rather than local hospital systems was also based on the fact that follow up data would not be available from local hospital systems for patients who are repatriated to other hospitals.

A major limitation is that we had no data on medications, either at hospital discharge or during follow up. Medications prescribed at discharge should, in principle, have been easy to obtain from participating hospitals; however, none of the hospitals had electronic pharmacy records that could be linked by a common patient identifier. Medications (either newly initiated or changes to existing prescriptions) could not be obtained during follow up because there are no medication records in any of the hospital episode datasets. There was consensus that initiation, withdrawal or changes in medication represented an important change in management for all our patient subgroups [10], in particular patients with LV thrombus for whom anticoagulation therapy is the main change in management. This limitation should be overcome in the near future with the increasing implementation and use of hospital electronic prescribing (EP) systems [15]. A potential solution to the lack of medications data during follow up is linkage of data collected from hospitals and hospital episode data with data from the Clinical Practice Research Datalink (CPRD), a primary care dataset that has detailed information on medications (https://www.cprd.com/). Only a subset of GP practices (6%) contribute data to CPRD, so medications data would only be available for a proportion of patients in a registry.

Other changes in management (e.g. additional diagnostic tests) were likely underestimated because a large proportion of diagnostic tests are performed on an outpatient basis, and some tests, such as bedside echocardiography, are not recorded in the admitted patient care dataset. Although we could identify the frequency of outpatient appointments under the cardiology specialty (and could have done so for cardiac surgery or any other related specialty), we could not identify the tests themselves because test codes are not well recorded in outpatient datasets.

Our approach to constructing a proxy outcome reflects our original concept and appears to be feasible when the important changes in management are hypothesised to cause a change in the same direction in all events / activities included in a composite outcome for a subgroup. However, through the consensus process [10], it became apparent that some of the hypothesised changes in management arising from CMR would not necessarily result in a unidirectional change in the frequency or rate of an activity. In several instances, an important change in management was more appropriate targeting of a treatment activity to the patients who were most likely to benefit, without necessarily changing the overall frequency of the activity (potentially constrained in publicly-funded health services). For example, in patients with MVD, CMR may result in better (i.e. more cost-effective) targeting of additional revascularisation. In these situations, a simple evaluation of the frequency or rate of a proxy outcome would not detect any value of CMR to patients and the NHS. It is also worth noting that some hospital activities representing the important changes in management we identified (e.g. time to outpatient appointments and additional diagnostic tests) will be complicated by local NHS driven issues which may obscure any CMR test related differences.

Although the “concept” of creating a composite outcome reflecting changes in care management can be applied to any imaging technique for any disease condition, our composite outcome was developed from a consensus process with cardiologists which identified the changes in management resulting from CMR relevant to patients activating the PPCI pathway. The outcome cannot therefore be applied to other imaging techniques, even within the same population, since it relies on CMR being able to identify specific complications from the myocardial infarction (MI) that would not be identifiable by other imaging techniques (at least not in all relevant patient subgroups).

## Conclusions

We conclude that it is feasible to ascertain events reflecting important changes in management, their frequencies and times to first event from HES/PEDW datasets. One important changes in management (change in medication) could not be identified from HES/PEDW admission datasets or directly from hospitals; this limitation should disappear as the NHS implements more electronic systems. HES data are generally regarded as reliable and have been used to identify most of the events/activities we required [11–14]. Further work is required to validate the reliability of HES/PEDW codes for identifying patient subgroups (OHCA and LV thrombus) and the key events representing the important changes in management (for example, by comparing with local hospital data, where available) before use in a main registry. Also, further work to derive a weighting algorithm for each patient subgroup is required before attempting to combine the events in a composite endpoint. Such an endpoint would need a careful assessment of the effects on its single components and their correlations, as the observed effect of the composite does not necessarily reflect the effects of the single components and will also require different weighting algorithms for different patient subgroups.

Our composite primary outcome will not be used in a large registry (as we had anticipated when we conceived the study) because the registry itself has proved not to be feasible at present due to limitations on data availability (e.g. exposure to tests) and consent (e.g. conventional ways of obtaining consent are too time consuming for a registry with no primary data collection [9]). However, this does not invalidate the concept of creating a composite outcome that reflects changes in management as a result of having an imaging test.

## Additional file

Additional file 1: ICD-10 diagnosis and OPCS procedure codes used to identify patient subgroups and changes in management in hospital episode data. (DOCX 16 kb)

## Data Availability

Anonymised individual patient data (excluding hospital episode/vital status linked data) can be made available for secondary research, conditional on assurance from the secondary researcher that the proposed use of the data is compliant with the MRC Policy on data preservation and sharing regarding scientific quality, ethical requirements and value for money. We are prevented from sharing hospital episode data/vital status data under our data sharing agreement with NHS Digital. Please contact maria.pufulete@bristol.ac.uk to discuss any data requests.

## Abbreviations

A&E: Accident and Emergency
APC: Admitted patient care
CABG: Coronary artery bypass grafting
CMR: Cardiac magnetic resonance
CPRD: Clinical Practice Research Datalink
CRT: Cardiac resynchronisation therapy
ECHO: Echocardiography
ED: Emergency Department
GP: General practitioner
HES: Hospital Episode Statistics
ICD: Implantable cardioverter defibrillator
ICD: International Classification of Diseases
IQR: Interquartile range
IVUS: Intravascular ultrasound
LV: Left ventricular
MACE: Major adverse cardiac-related events
MVD: Multivessel disease
NHS: National Health Services
OHCA: Out of hospital cardiac arrest
OPCS: Office of Population Censuses and Surveys Classification of Interventions and Procedures
PEDW: Patient Episode Database Wales
PET: Positron emission tomography
PPCI: Primary percutaneous coronary intervention
STEMI: ST-elevation myocardial infarction
