# Correction to: Feasibility of identifying important changes in care management resulting from cardiovascular magnetic resonance (CMR) using hospital episode data in patients who activate the primary percutaneous coronary intervention (PPCI) pathway

**DOI:** 10.1186/s12874-019-0844-3

**Published:** 2019-10-15

**Authors:** Maria Pufulete, Jessica Harris, Stephen Dorman, Lynn Cook, Chiara Bucciarelli-Ducci, John P. Greenwood, Richard Anderson, Rachel Brierley, Barnaby C. Reeves

**Affiliations:** 1 Clinical Trials and Evaluation Unit, University of Bristol, Level 7, Bristol Royal Infirmary, Queen’s Building, Bristol, UK; 2 NIHR Bristol Cardiovascular Research Unit, Bristol Heart Institute, University of Bristol, Bristol, UK; 3 Department of Information Management and Technology, University Hospitals Bristol NHS Foundation Trust, Bristol, UK; 4 Multidisciplinary Cardiovascular Research Centre and Leeds Institute of Cardiovascular and Metabolic Medicine, University of Leeds, Leeds, UK; 5 University Hospitals of Wales, Heath Park, Cardiff, UK

**Correction to: BMC Med Res Methodol (2019) 19:116**

**https://doi.org/10.1186/s12874-019-0755-3**

In the original publication of this article [1], the author noted two corrections after publication.

1. There is a middle initial in John P. Greenwood.
2. An institution’s name in the Funding section should be changed from “NIHR Bristol Biomedical Research Unit for Cardiovascular Disease” to “NIHR Bristol Biomedical Research Centre”.

The original article has been corrected.

The authors apologize for the inconvenience caused to our readers.
