# Supplementary material for: Feasibility of identifying important changes in care management resulting from cardiovascular magnetic resonance (CMR) using hospital episode data in patients who activate the primary percutaneous coronary intervention (PPCI) pathway
Source: BMC Med Res Methodol. 2019 Jun 6;19:116. doi: 10.1186/s12874-019-0755-3 (PMC6554929; doi:10.1186/s12874-019-0755-3)
Supplement: Supplementary file 1 — ICD-10 diagnosis and OPCS procedure codes used to identify patient subgroups and changes in management in hospital episode data. (DOCX 16 kb) [file 12874_2019_755_MOESM1_ESM.docx]

**OPCS procedure and ICD-10 diagnosis codes**

|  | |
| --- | --- |
| **ICD-10 Diagnosis codes: OHCA** | |
| I46 | Cardiac arrest |
| **ICD-10 Diagnosis codes: LV thrombus after PPCI** | |
| I23.6 | Thrombosis of atrium, auricular appendage and ventricle as current complications following acute myocardial infarction |
| **ICD-10 Diagnosis codes: Takotsubo cardiomyopathy** | |
| I42.8 ***AND*** | Other cardiomyopathies |
| F43.8 | Other reactions to severe stress |
| **ICD-10 Diagnosis codes: Myocarditis** | |
| I51.4 | Myocarditis, unspecified |
| **ICD-10 Diagnosis codes: Pericarditis** | |
| I30 | Acute pericarditis |
| I31.0 | Chronic adhesive pericarditis |
| I31.1 | Chronic constrictive pericarditis |
| I31.9 | Disease of pericardium, unspecified |
| I32.0 | Pericarditis in bacterial diseases classified elsewhere |
| I32.1 | Pericarditis in other infectious and parasitic diseases classified elsewhere |
| I32.8 | Pericarditis in other diseases classified elsewhere |
| I01.0 | Acute rheumatic pericarditis |
| I02.0 | Rheumatic chorea with heart involvement |
| I09.2 | Chronic rheumatic pericarditis |
| **ICD-10 Diagnosis codes: Endocarditis** | |
| I33.9 | Acute endocarditis, unspecified |
| **ICD-10 Diagnosis codes: Coronary spasm** | |
| I20.1 | Angina pectoris with documented spasm |
| **OPCS Procedure codes: ECHO** | |
| K58.5 | Transluminal intracardiac echocardiography |
| U20.1 | Transthoracic echocardiography |
| U20.2 | Transoesophageal echocardiography |
| U20.3 | Intravascular echocardiography |
| U20.4 | Epicardial echocardiography |
| U20.5 | Stress echocardiography |
| **OPCS Procedure codes: Single photon emission computed tomography** | |
| U21.4 | Single photon emission computed tomography NEC |
| **OPCS Procedure codes: PET scan** | |
| U10.4 | Myocardial positron emission tomography |
| U21.3 | Positron emission tomography NEC |
| U36.2 | Positron emission tomography with computed tomography NEC |
| **OPCS Procedure codes: IVUS** | |
| K51.2 | Intravascular ultrasound of coronary artery |
| L726 | Intravascular ultrasound of artery NEC |
| **OPCS Procedure codes: Pressure wire** | |
| K63.4- K63.6 ***AND*** | Coronary arteriography |
| K51.8 ***AND*** | Other specified diagnostic transluminal operations on coronary artery |
| Y44.2 ***AND*** | Monitoring of pressure in organ NOC |
| Y53.- | Approach to organ under image control |
| **OPCS Procedure codes: Radionuclide angiocardiography** | |
| U10.5 | Radionuclide angiocardiography |
| **OPCS Procedure codes: Computed tomography angiography** | |
| U10.2 | Cardiac computed tomography angiography |
| **OPCS Procedure codes: Implantation of CRT** | |
| K60.7 | Implantation of intravenous biventricular cardiac pacemaker system |
| K61.7 | Implantation of biventricular cardiac pacemaker system |
| K59.6 | Implantation of cardioverter defibrillator using three electrode leads |
| **OPCS Procedure codes: Implantation of ICD** | |
| K59 | Cardioverter defibrillator introduced through the vein |
| K72 | Other cardioverter defibrillator |
| **OPCS Procedure codes: PCI** | |
| K49 | Transluminal balloon angioplasty of coronary artery |
| K50 | Other therapeutic transluminal operations on coronary artery |
| K75 | Percutaneous transluminal balloon angioplasty and insertion of stent into coronary artery |
| **OPCS Procedure codes: CABG** | |
| K40 | Saphenous vein graft replacement of coronary artery |
| K41 | Other autograft replacement of coronary artery |
| K42 | Allograft replacement of coronary artery |
| K43 | Prosthetic replacement of coronary artery |
| K44 | Other replacement of coronary artery |
| K45 | Connection of thoracic artery to coronary artery |
| K46 | Other bypass of coronary artery |
